# Introducing low-quality feedstocks in bioethanol production: efficient conversion of the lignocellulose fraction of animal bedding through steam pretreatment

**DOI:** 10.1186/s13068-019-1558-9

**Published:** 2019-09-10

**Authors:** Miguel Sanchis-Sebastiá, Borbála Erdei, Krisztina Kovacs, Mats Galbe, Ola Wallberg

**Affiliations:** Department of Chemical Engineering, Lund University, P.O. Box 124, 221 00 Lund, Sweden

**Keywords:** Animal bedding, Steam pretreatment, Bioethanol, Response-surface modeling

## Abstract

**Background:**

Animal bedding remains an underutilized source of raw material for bioethanol production, despite the economic and environmental benefits of its use. Further research concerning the optimization of the production process is needed, as previously tested pretreatment methods have not increased the conversion efficiency to the levels necessary for commercialization of the process.

**Results:**

We propose steam pretreatment of animal bedding, consisting of a mixture of straw and cow manure, to deliver higher ethanol yields. The temperature, residence time and pH were optimized through response-surface modeling, where pretreatment was evaluated based on the ethanol yield obtained through simultaneous saccharification and fermentation of the whole pretreated slurry. The results show that the best conditions for steam pretreatment are 200 °C, for 5 min at pH 2, at which an ethanol yield of about 70% was obtained. Moreover, the model also showed that the pH had the greatest influence on the ethanol yield, followed by the temperature and then the residence time.

**Conclusions:**

Based on these results, it appears that steam pretreatment could unlock the potential of animal bedding, as the same conversion efficiencies were achieved as for higher-quality feedstocks such as wheat straw.

## Background

Lignocellulosic ethanol is still too expensive to compete with fossil fuels on the commercial scale, due to its relatively high production cost, the main contributions being the cost of the feedstock and the capital cost [1]. In fact, the cost of the feedstock can represent as much as one-third of the total production cost [2], and the use of new feedstocks with zero or negative value will be required to achieve cost competitiveness [1]. Animal manure is one example of such a low-value feedstock, and ethanol production could offer a way of valorizing a biomass source that is usually lost otherwise [3]. The use of this material as feedstock would reduce the cost of the raw material in the ethanol production process and, at the same time, alleviate the problem of waste disposal, which would counterbalance the environmental impact of the ethanol production process [3]. Animal manure is thus an attractive feedstock from both the economic and environmental perspectives.

In spite of these advantages, animal manure has been little explored as a resource in bioenergy production [4], although a few studies have been carried out on ethanol production from animal manure. For example, Gomaa et al. concluded that this feedstock had potential as a raw material for biogas and bioethanol production [5]. However, the ethanol yields obtained in their study were low, and they pointed out the need for further research to optimize the production process. Some studies have found that a pretreatment step is necessary to enhance the release of sugars from animal manure, and thus improve the ethanol yield [6, 7]. This would be especially the case for animal manure with a high fiber content, such as farmyard cow manure [8]. The effect of acid concentration, pretreatment time and cellulase dosage in pretreatment involving acid hydrolysis followed by enzymatic hydrolysis, on the fermentability of farmyard cow manure has been studied by Vancov et al. [9]. They reported the highest ethanol yield to date of 55% of the theoretical maximum based on the glucose in the raw material. Although this is a significant improvement compared to previous yields of 20% [8], the authors stated that further development was needed to realize the potential of cow manure as a feedstock for bioenergy production [9].

This study was carried out to investigate the effect of steam pretreatment instead of acid hydrolysis. This technology would reduce both the environmental impact and the cost of pretreatment [10], and we hypothesized that higher ethanol yields could be obtained from the fibrous fraction of animal bedding, which is a mixture of straw and cow manure. To validate this hypothesis, we tested several operating conditions in a steam pretreatment reactor to identify the maximum ethanol yield obtainable and compared the results with the yields obtained previously from similar materials. In addition, we modeled the effect of temperature, residence time and pH in the pretreatment step on the ethanol yield to identify trends that explained the results obtained, which could be extrapolated to the design of other processes based on similar materials.

## Results and discussion

### Raw material composition

Table 1 gives the composition of the raw material and the fiber fraction after washing in a concrete mixer with deionized water at room temperature. Fermentable carbohydrates accounted for almost 40% of the dry mass of the unwashed bedding, which proves that this material could become an important source of substrate for bioethanol production. Moreover, 30% of the dry mass of the unwashed bedding (the organic part of the manure) could potentially be used as a substrate for biogas production, which illustrates the high potential of animal bedding as a resource for bioenergy production, since approximately 70% of its dry mass could be used for this purpose.

**Table 1:** Composition of the animal bedding before and after washing

Content (%DM bedding)	Animal bedding	Fiber after washing
Manure	43.4	10.5
Organic matter	29.7	7.2
Inorganic matter	13.7	3.3
Fiber	56.6	89.5
Glucan	24.1	38.1
Xylan	11.6	18.3
Galactan	0.5	0.8
Arabinan	1.2	1.9
Mannan	0.6	0.9
Lignin	11.8	18.7
Extractives	4.2	6.7
Ash	1.9	2.9

The composition of the animal bedding presented in this study is similar to that reported by Bona et al. [8]. However, the manure content is lower, and the fermentable carbohydrate content is higher, than those reported by Vancov et al. [9], while the opposite is true compared with the composition reported by Chen et al. [6]. This variation can be expected, as the composition of such material is affected by many factors, such as the kind and number of animals, their diet, animal housing and time spent in the stable [11].

Washing reduced the manure content of the material from 43 to 10% (Table 1), as the average washing efficiency was 75.8% with a standard deviation of 3.6%. After washing, the material has a composition very similar to that of wheat straw [12, 13], despite the fact that the washed fiber still contains a small fraction of manure. Although the residual manure could give rise to the Maillard reaction during pretreatment [14], it can be expected that the washed fiber would behave similarly to wheat straw during steam pretreatment, as the materials have very similar compositions.

### Pretreatment

The fiber fraction obtained after washing the material with water was pretreated with steam and subsequently analyzed before its conversion to bioethanol. Rather than discussing the complete composition of all the materials, which can be found in Additional file 1, the intention of this section is to validate the data through checking its consistency with the chemistry of steam pretreatment reported previously in the literature, and comparing the results with those obtained when performing steam pretreatment on wheat straw.

**Fig. 1:**
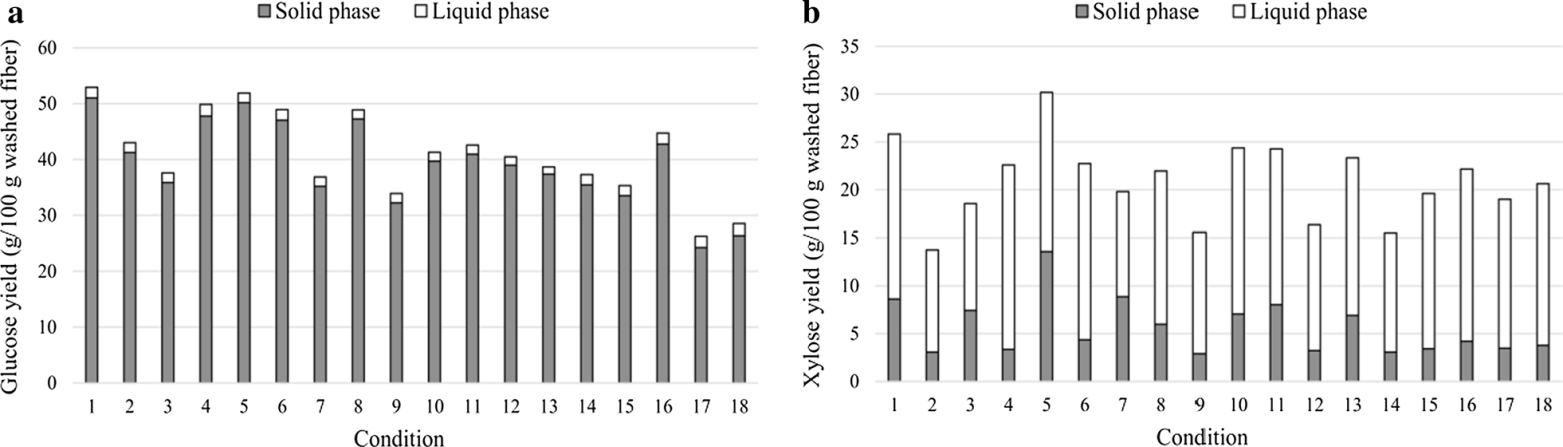
Yield of glucose (**a**) and xylose (**b**) for each set of conditions in the steam pretreatment of the fiber fraction of animal bedding

The fiber fraction of the pretreated materials contained 43–59% glucan, 4–14% xylan and 32–38% lignin, depending on the pretreatment conditions, while the liquid fraction contained mainly xylose, at concentrations between 21 and 41 g/L, and only minor amounts of glucose and other sugars. This implies that cellulose and lignin remained mostly in the solid phase after pretreatment, while hemicelluloses were solubilized (Fig. 1), which is consistent with the chemistry of pretreatment performed at low pH [15]. Moreover, these compositions are similar to those reported for steam-exploded wheat straw in previous studies [16, 17], which indicates that washed animal bedding behaves similarly to wheat straw during steam pretreatment.

**Fig. 2:**
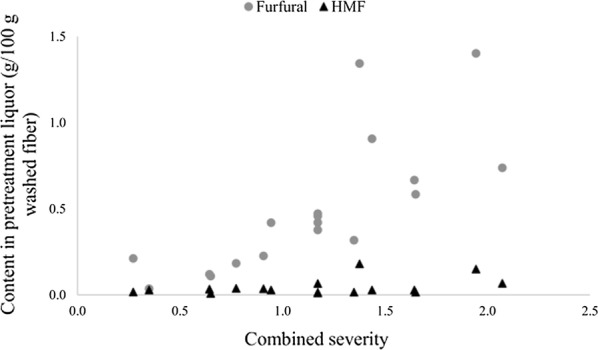
Furfural and HMF contents in the pretreatment liquor after steam pretreatment of the fiber fraction of animal bedding

A fraction of the sugars released during pretreatment was degraded into other by-products, such as furfural and hydroxymethylfurfural (HMF); this effect became more pronounced as the severity of the pretreatment was increased (Fig. 2). This is consistent with the conclusions about carbohydrate degradation during steam pretreatment presented by Li et al. [18]. The generation of by-products during steam pretreatment does not follow the same pattern as for wheat straw, as the furfural production was higher and the HMF production lower than the results reported by Ballesteros et al. [19]. It thus appears that, although sugars are recovered in a similar fashion, carbohydrate degradation during steam pretreatment differs between animal bedding and wheat straw, possibly due to the presence of residual manure in the material that can trigger various degradation mechanisms, such as the Maillard reaction [14].

Despite the differences in by-product generation, the amount of furfural generated during pretreatment is not high enough to compromise the efficacy of fermentation, since furfural concentrations over 3 g/L are necessary to affect the performance of *S.* cerevisiae [20]. Thus, by-product formation does not appear to be critical when pretreating the fiber fraction of animal bedding, as the concentrations of the by-products obtained are not toxic to the fermenting microorganism. However, this may be a problem when using pretreatment techniques that produce a material with a higher dry matter content, as the resulting inhibitor concentrations may be higher.

### Simultaneous saccharification and fermentation

The material steam pretreated at each of the conditions tested was converted into ethanol through simultaneous saccharification and fermentation (SSF), and the yield obtained in each case is given in Table 2. The ethanol yield ranged from 36.3 to 69.3% depending on the pretreatment conditions, and the maximum error between duplicates, obtained for conditions 2 and 8, was 0.03 g ethanol/g glucose in the washed fiber.

**Table 2:** Ethanol yield after SSF for each of the conditions tested in the steam pretreatment

Condition	Ethanol yield (g/g)	Ethanol yield (% max theoretical)
1	0.302	59.1
2	0.279	54.7
3	0.228	44.6
4	0.354	69.3
5	0.253	49.5
6	0.318	62.3
7	0.185	36.3
8	0.284	55.7
9	0.262	51.3
10	0.215	42.2
11	0.222	43.4
12	0.288	56.4
13	0.202	39.5
14	0.301	59.0
15	0.248	48.6
16	0.263	51.5
17	0.204	42.7
18	0.300	58.9

The maximum yield obtained in this study (69.3% for condition 4) was higher than those previously reported for acid hydrolysis pretreatment, although the results are not strictly comparable since the methods used to perform the biological steps were not the same. For example, a yield of 55.3% has previously been achieved using acid hydrolysis [9], while a lower yield of 22.2% was reported in another study using the same technology [8]. The results obtained with steam pretreatment also compare well to those from other technologies based on high pH, such as the NaOH pretreatment applied by You et al. [21], with which the authors achieved a yield of 39.9%. The outcome is also favorable when compared to radically different technologies, such as pretreatment by anaerobic digestion followed by NaOH treatment, proposed by Yue et al. [22]. They obtained a highly digestible fiber, leading to high enzymatic hydrolysis and fermentation yields (90% and 72%, respectively) but, based on their mass balances, there is a cellulose loss of 24% during NaOH treatment, which lowers the combined sugar yield based on the original fiber to 46.7%. Thus, it seems that our initial hypothesis is valid, and steam pretreatment allows higher yields to be obtained than with previously tested technologies. However, to confirm the hypothesis irrefutably, it would be necessary to evaluate the performance of the different technologies with the same methodology to obtain results that can be directly compared.

The limitation on the ethanol yields obtained from farmyard cow manure could be attributed to the relatively low recoveries achieved in the pretreatment step, as the hydrolysis and fermentation yields are usually within an acceptable range. For example, acid hydrolysis provided 79% sugar recovery [9], which is very similar to that obtained using NaOH pretreatment [22]. This means that the excellent sugar recovery that characterizes steam pretreatment [18] might be the reason why this technology enables higher conversion efficiencies (approximately 90–100% recoveries were obtained in this study).

The maximum yield in our study is also in the same range as those reported for ethanol production from steam-exploded wheat straw [19, 23–26], which indicates that fractionation with water (i.e., washing) followed by steam pretreatment allows the same conversion efficiencies to be achieved as for higher-quality residues. This implies that the technology proposed in this study could help to unlock the potential of cow manure as a resource for bioenergy production, since the same conversion efficiencies can be achieved, but at a reduced feedstock price.

### Modeling and optimization

#### Model development and validation

We developed a model that relates the ethanol yield to the operational parameters in the pretreatment step using multiple linear regression (Eq. 1). The model was developed based on the coded variables, which implies that the coefficients in the model are a measure of the significance of each of the terms included in the model [27], i.e., a larger coefficient in Eq. 1 means that the term has a greater influence on the response (ethanol yield).

1 $$\begin{aligned} Y_{\text{EtOH}} \left( \% \right) = & 50.8 + 4.1T + 3.0t - 4.8{\text{pH}} + 0.4T^{2} + 0.6t^{2} - 0.3{\text{pH}}^{2} - 2.5T \cdot t + 1.5T \\ \cdot {\text{pH}} + 4.5t \cdot {\text{pH}} + 2.8T \cdot t \cdot {\text{pH}} . \\ \end{aligned}$$

To validate the model, the variance was disaggregated into several fractions through an ANOVA analysis (Table 3). The variance due to the residuals, i.e., the variance not explained by the model, can be used to calculate the value of *R*^2^ for the model, which was 0.758. Although this value may seem low, *R*^2^ is not sufficient to evaluate the goodness of fit of a model, since it does not consider the degrees of freedom, and contains no information on the source of the error in the prediction [28]. In fact, when considering the degrees of freedom using a test for the significance of the regression, it can be said that there is an 85% probability (*p* = 0.1405) that at least one of the coefficients is different from zero or, in other words, that the model is significant, which is acceptable for this kind of system.

**Table 3:** ANOVA for the model developed to relate ethanol yield to the operational parameters in the pretreatment step

Source	Degrees of freedom	Sum of squares	Mean square
Total	18	48,853.0	2714.1
Mean	1	47,535.0	47,535.0
Corrected	17	1318.6	77.6
Factor effects	10	999.1	99.9
Residuals	8	319.5	39.9
Lack of fit	4	183.5	45.9
Purely experimental uncertainty	3	136.0	45.3

ANOVA analysis deals with this apparent inconsistency through further separating the variance due to the residuals into: (i) the variance due to the lack of fit, which corresponds to that originating from bad fitting of the coefficients, and (ii) the variance due to experimental uncertainty. Based on these variances, a test for the lack of fit was applied, and the result showed that there was only a 66% probability (*p* = 0.3394) that the lack of fit is significant, which is low compared to the usually applied 95% confidence level. It can then be said that the effects of temperature, residence time and pH on the ethanol yield are correctly fitted, even though the predictive power of the model is low due to the relatively large experimental errors and possible uncontrolled factors.

The practical meaning of these results in terms of the ethanol production process is that the operating conditions in the steam pretreatment determine the ethanol yield of the process to a large extent, but not completely. This implies that the ethanol yield cannot be predicted based solely on the conditions chosen for pretreatment. Small fluctuations can be expected due to random errors in the overall process, and larger errors may arise from uncontrolled changes in factors deemed constant, such as the composition of the feedstock, the activity of the enzymes and the vitality of the yeast.

#### Size of the effects

The influence of the pretreatment variables on the ethanol yield was further investigated by performing a test for a set of parameters to determine the significance of each part of the model. The test showed that there is a 98% (*p* = 0.0159) probability that the linear terms are significant, while this probability is only 36% (*p* = 0.6426) for the quadratic terms, and 76% (*p* = 0.2343) for the interaction terms. The reason for this is that, of the 75% variance explained by the model, 69% is explained by the linear terms, 2% by the quadratic terms and 29% by the interaction terms. From this it can be seen that the pretreatment variables have a strong linear effect on the ethanol yield in the range studied, and that there are relevant interactions between them, while the curvature due to quadratic effects is minimal.

It is possible that the small size of the quadratic terms is a result of the range of conditions included in the study, which is relatively small, and not necessarily because these effects do not exist. A function with a curvature may appear linear when analyzed over a small range and, therefore, larger quadratic effects could have been found if the pretreatment variables had been studied over larger ranges. However, a larger range in the pretreatment variables would lower the precision in the fitting of the model [28, 29]. Thus, the model presented provides a more accurate representation of the effects near the optimal operating conditions, although it may not be valid for extreme conditions at much lower or higher combined severity, as defined by Chum et al. [30], than those tested in this study.

The curvature in the model is the result of interaction effects, which means that increasing the severity by changing one of the variables limits the severity that can be achieved through changing the others. This result is consistent with the fact that the optimal pretreatment severity is governed by carbohydrate degradation [18], and also with the results reported by Vancov et al. [9], where interactions between the pretreatment variables were also found for acid hydrolysis pretreatment of cow manure. The existence of these interactions makes the prediction of the outcome of pretreatment more complex, because the pretreatment variables are not completely interchangeable, i.e., different results could be obtained when increasing the severity by raising the temperature than by increasing the residence time.

**Fig. 3:**
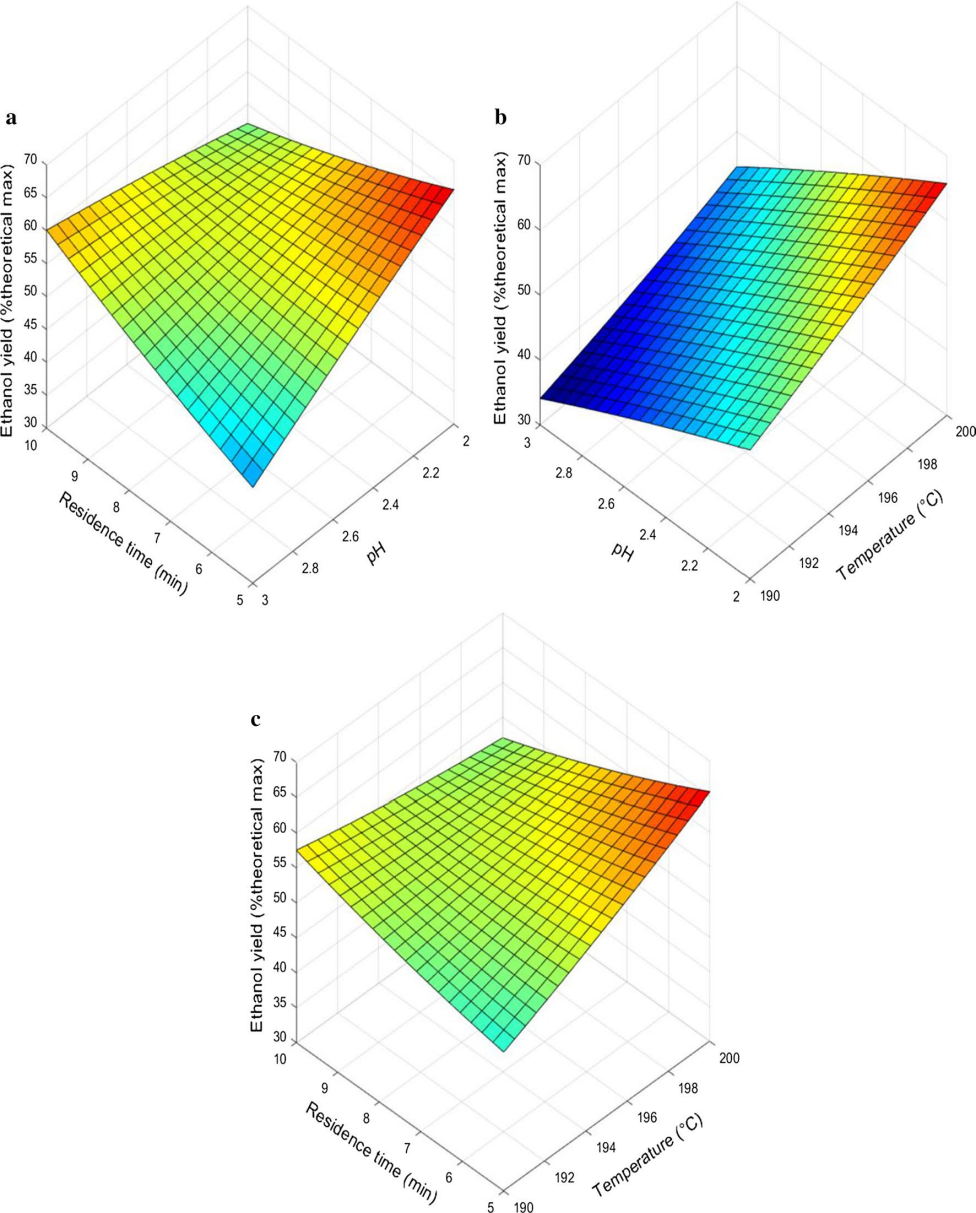
Response surfaces at 200 °C (**a**), 5 min (**b**) and pH = 2 (**c**), based on the predictions of the model

To understand which of the pretreatment variables is more significant, it was necessary to analyze the model as a whole, rather than for just a set of parameters. Response surfaces were used for this purpose, in particular three surfaces at the value of each pretreatment variable giving the best conditions tested, i.e., *T* = 200 °C, t = 5 min and pH = 2 (Fig. 3). The more significant a variable is, the more it can compensate for suboptimal values of the other variables; therefore, the change in ethanol yield represented by the response surface can be used as an indication of the significance of the variable. For example, when the time is at its optimal value but the other variables are not, the ethanol yield decreases to 35% (Fig. 3b), but in the analogous situation for the pH, the yield is only reduced to 55% (Fig. 3c), which indicates that the pH has a greater influence on the yield than the residence time. Based on this, it can be said that the residence time influences the ethanol yield to a much lower extent than the temperature and the pH, which have a similar degree of influence, although that of the pH is slightly higher.

#### Model-based optimization

Based on the optimization of the model, the best conditions for steam pretreatment are 200 °C, for 5 min, at pH 2, which is one of the tested conditions, so no further validation was required. The optimal condition found for animal bedding was the same as that previously reported for wheat straw [19, 24], which shows that the time the bedding is in the stable does not help overcome the recalcitrance of the material, as the same severity is needed in its pretreatment.

Due to the relatively low predictive power of the model, the optimum may instead be at 190 °C, for 10 min, at pH 2. Other authors have reported that these two conditions gave very similar results in terms of ethanol yield in the subsequent biological processes [19], and it is therefore difficult to reach a level of accuracy that allows differentiation between them. In spite of this, the optimum does not lie outside the tested range, since the best yields were not obtained for either the lowest or the highest severity, although a more accurate estimate might be obtained through further experimentation in the vicinity of the reported optimum.

## Conclusions

Design of experiments together with response-surface modeling was used to optimize the pretreatment conditions to maximize the ethanol yield from animal bedding. The optimal conditions were 200 °C, for 5 min, at pH 2, at which an ethanol yield of 69.3% was obtained. The yield obtained when using steam pretreatment was higher than that obtained with other pretreatment technologies previously tested and was in the same range as that for steam-exploded wheat straw. This means that steam pretreatment may provide a means of unlocking animal bedding as a resource for bioenergy production, as the same conversion efficiencies can be obtained as for higher-quality feedstocks.

Further analyses of the model showed that pH has the greatest influence on the ethanol yield, followed closely by the temperature, and that residence time has considerably less influence. Although the effects were properly fitted, the predictive power of the model may be low due to the high experimental variability, and the possible existence of uncontrolled factors. This implies that, in an ethanol production process based on animal bedding, it would not be possible to predict the yield of the process based only on the pretreatment conditions, although they determine it to a large extent.

## Materials and methods

### Animal bedding collection and preparation

Animal bedding was collected from a dairy farm at Lille Skensved, a small town close to Køge, in Denmark. The barn is approximately 600 m^2^, has a rectangular shape, hosts 150 dairy cows in a loose house regime, and approximately 500 ton of straw is used per year as bedding. Samples were collected from 13 different positions in the barn and stored frozen until further use, according to previous recommendations [31].

After sample collection, the animal bedding from each of the sampling positions was washed with deionized water to separate the manure from the straw. Washing was performed by mixing 4 kg of animal bedding (approximately 1 kg dry animal bedding) with 10 L of deionized water at room temperature in a concrete mixer for 2 min. The material was subsequently pressed in a filter press to remove the liquid, which contained most of the manure.

After washing, a subsample of the washed animal bedding was taken from the material collected at each of the 13 sampling positions after thorough mixing of the material in the concrete mixer. The 13 subsamples were then mixed to produce an average sample that is representative of the whole barn. This average material was used in the pretreatment and fermentation experiments.

### Steam pretreatment

The washed animal bedding was impregnated with sulfuric acid by soaking in a dilute sulfuric acid solution (0.3–0.6 wt% depending on the pretreatment conditions) for 1 h. Soaking was performed at a solid-to-liquid ratio of 1:20, and sulfuric acid was added progressively until the desired pH was reached. Different pH levels, from 1.6 to 3.4, were tested according to the experimental design described in Sect. “Experimental design and statistical analysis”. The material was pressed in a filter press at 13 bar to remove the liquid, and the soaked fiber was left overnight at room temperature in a sealed container prior to steam pretreatment.

The soaked fiber was then subjected to steam pretreatment in a 10 L reactor (Process & Industriteknik AB, Kristianstad, Sweden), which has been described elsewhere [32]. Steam pretreatment was performed at various conditions, from 186 to 204 °C, for 3–12 min, according to the experimental design described in Sect. “Experimental design and statistical analysis”. At each condition, 600 g dry soaked fiber was pretreated and the pretreated materials were stored at 4 °C before further use for analysis or experiments.

### Simultaneous saccharification and fermentation

SSF experiments were performed on the whole pretreated slurry in 2 L Labfors bioreactors with a working weight of 1 kg. Prior to running the experiments, the fermenters with the slurry were sterilized (after correcting the pH of the material to 5). A water-insoluble solid (WIS) load of 8%, Cellic CTec2 (Novozymes, Denmark) enzyme cocktail at a load of 0.05 g enzyme/g WIS (which corresponds approximately to 10 FPU/g WIS) and Ethanol Red (Lesaffre Advanced Fermentations, France) yeast at a dry weight concentration of 3 g/L were used during the experiments. Due to severe mixing difficulties at the start of SSF, mixing at 400 rpm was applied 1 h after adding the enzymes and the yeast, when the material had become sufficiently liquefied to be mixable. SSF was performed at 35 °C and the pH was maintained at 5 through the automatic addition of 10% NaOH solution. The SSF media were supplemented with 0.5 g/L (NH_4_)_2_PHO_4_, 0.025 g/L MgSO_4_, 1 g/L yeast extract and, to avoid the risk of infection, 10 mg/L streptomycin and 10,000 U/L penicillin. All the SSF experiments were performed in duplicate.

Samples obtained from the SSF experiments were centrifuged in 2 mL Eppendorf tubes at 13,000 rpm for 5 min. The supernatant was filtered through 0.2 μm syringe filters (GVS North America, Sanford, USA) and stored at − 20 °C prior to high-performance liquid chromatography (HPLC) analysis. Ethanol, organic acids and other by-products were analyzed using a Shimadzu LC-20 AD HPLC system equipped with a Shimadzu RID 10A refractive index detector (Shimadzu Corporation, Kyoto, Japan). The chromatography column used was an Aminex HPX-87H, with a Cation-H Bio-Rad Micro-Guard column (Bio-Rad Laboratories, Hercules, United States) at 50 °C, and a 5 mM sulfuric acid solution was used as eluent at a flow rate of 0.5 mL/min.

### Compositional analysis

Animal bedding is considered to be a mixture of two components, manure and straw, and the manure content is assumed to be equal to the mass removed after ten washing cycles [33]. The manure was further analyzed by incinerating a sample at 575 °C for 3 h to determine the organic matter content, and the inorganic matter content was calculated as the difference between the total solids and the organic matter content. The straw was analyzed following the protocols from the National Renewable Energy Laboratory (NREL) [34–37].

The manure content of the washed animal bedding was calculated as the product of the manure content in the native material and one minus the average washing efficiency of the 13 samples (Eqs. 2 and 3). The rest of the composition of the washed animal bedding was calculated assuming that the composition of the manure and the straw remained constant during washing and are therefore the same as that in the native material.

2 $${\text{Manure}}\left( {\% {\text{TS}}} \right) = {\text{manure}}_{\text{native}} \cdot \left( {1 - {\text{wash}}_{\text{eff}} } \right) ,$$

3 $${\text{Wash}}_{\text{eff}} = \frac{{\sum \frac{{M_{\text{liquid}} \cdot{\text{TS}}_{\text{liquid}} }}{{{\text{manure}}_{\text{native}} \cdot M_{\text{bedding}} }}}}{{N_{\text{samples}} }} ,$$

where manure_native_ is the manure content in the native material, expressed as %TS; *M*_liquid_ the mass of the expressed liquid after washing; TS_liquid_ the total solid content of the expressed liquid after washing; *M*_bedding_ the dry mass of the animal bedding washed; and *N*_samples_ the number of samples that were washed (13 in this study).

The WIS content of the pretreated materials was determined using the non-wash method described by Weiss et al. [38]. The structural carbohydrates and lignin content of the solid fraction and the composition of the liquid fraction were analyzed following NREL protocols [37, 39]. Monomeric sugars in the liquid fraction were analyzed using the HPLC system described above, using an Aminex HPX-87P chromatography column with a De-Ashing Bio-Rad Micro-Guard column at 85 °C, using reagent-grade water as eluent at a flow rate of 0.6 mL/min. Pretreatment by-products in the liquid fraction were analyzed using the same HPLC system, chromatographic column, and conditions as described in “Simultaneous saccharification and fermentation”.

Sugar samples generated during the analyses of structural carbohydrates and lignin were analyzed using high-performance anion-exchange chromatography coupled with pulsed amperometric detection. A Dionex system with a Carbo Pac PA1 column, a GP50 gradient pump and an AS50 autosampler were used. The flow rate was 1 mL/min, the temperature was 30 °C and the solutions used as eluents were: deionized water, 200 mmol/L NaOH, and 200 mmol/L NaOH mixed with 170 mmol/L sodium acetate.

### Yield calculations

The ethanol yield was calculated based on the total available glucose in the washed fiber, which corresponds to 1.11 times the amount of glucan in the fiber (due to the addition of water during hydrolysis). The yield is presented as g ethanol/g glucose in the raw material (washed fiber), and also as a percentage of the theoretical stoichiometric ethanol yield (0.51 g/g), which are the values used in the development of the model described in Sect. “Experimental design and statistical analysis”.

### Experimental design and statistical analysis

The effects of the three pretreatment variables, temperature (T), residence time (t) and pH during soaking (pH), on the ethanol yield were investigated using response-surface modeling. A spherical central composite design was chosen due to its improved performance [40], and four replicates were performed at the center point (195 °C, 7.5 min, pH 2.5), which was chosen based on previously reported optimal conditions for wheat straw [19]. The variables were coded to prevent scale effects from influencing the modeling. The coding was based on centering so that the zero value was assigned to the values of the variables at the center point, and the rest of the values were calculated based on the following conversion factors: 5 °C/coded unit, 2.5 min/coded unit and 0.5 pH units/coded unit. Table 4 gives the value of the variables in each of the 18 runs in both uncoded and coded units.

**Table 4:** Experimental design used to investigate the pretreatment step

Condition	Temperature (°C)	Residence time (min)	pH^a^	Coded units		
1	200	10	3	1	1	1
2	200	10	2	1	1	− 1
3	200	5	3	1	-1	1
4	200	5	2	1	-1	− 1
5	190	10	3	− 1	1	1
6	190	10	2	− 1	1	− 1
7	190	5	3	− 1	− 1	1
8	190	5	2	− 1	− 1	− 1
9	195	7.5	1.6	0	0	− 1.8
10	195	7.5	3.4	0	0	1.8
11	195	3	2.5	0	− 1.8	0
12	195	12	2.5	0	1.8	0
13	186	7.5	2.5	− 1.8	0	0
14	204	7.5	2.5	1.8	0	0
15	195	7.5	2.5	0	0	0
16	195	7.5	2.5	0	0	0
17	195	7.5	2.5	0	0	0
18	195	7.5	2.5	0	0	0

^a^pH in the impregnation step

An empirical model was constructed through multiple linear regression, as described previously by Brereton et al. [27] The model includes an intercept term, linear effects, quadratic effects and interaction terms (Eq. 4). The interaction terms account for the possibility that the value of one variable may influence the effect of another on the response [27]. For example, the effect of the pH may be different at low temperatures than at high temperatures.

4 $$\begin{aligned} Y_{\text{EtOH}} \left( \% \right) =\, & a_{0} + a_{1} T + a_{2} t + a_{3} {\text{pH}} + a_{4} T^{2} + a_{5} t^{2} + a_{6} {\text{pH}}^{2} + a_{7} T \cdot t + a_{8} T \cdot {\text{pH}} + a_{9} t \\ \cdot {\text{pH}} + a_{10} T \cdot t \cdot {\text{pH}} .\\ \end{aligned}$$

To evaluate the validity of the model, the value of *R*^2^ was complemented with further significance analyses based on the ANOVA methodology, as described elsewhere [28]. First, a test for the significance of the regression was performed to evaluate the effectiveness of the model as a whole, i.e., to determine whether at least one of the terms in the model was significant. Since this test offers no insight into which terms are significant, a test for a set of parameters was used to determine the significance of each part of the model (linear, quadratic and interaction), and to calculate their respective contributions to *R*^2^. Finally, a test for the lack of fit was performed to identify whether the terms in the model are fitted correctly. This test provides insight into the source of errors in the prediction, as it specifies whether the lack of fit originates from poor fitting of the model or from experimental error. All these statistical tests were performed according to the methodology described by Deming et al. [28].

All the calculations required for the development of the model and further statistical analyses were performed in MATLAB, and the probability density for the F function was calculated using the built-in MATLAB command *fpdf*.

## Supplementary information

**Additional file 1.** Composition of the pretreated materials at each of the conditions tested for the steam treatment.

## Data Availability

All data generated or analyzed during this study are included in this published article and its additional file.
